# Electronic *Lieb* lattice signatures embedded in two-dimensional polymers with a square lattice†

**DOI:** 10.1039/d3sc06367d

**Published:** 2024-03-12

**Authors:** Yingying Zhang, Shuangjie Zhao, Miroslav Položij, Thomas Heine

**Affiliations:** a Chair of Theoretical Chemistry, Technische Universität Dresden Bergstrasse 66 01069 Dresden Germany thomas.heine@tu-dresden.de; b Helmholtz-Zentrum Dresden-Rossendorf, HZDR Bautzner Landstr. 400 01328 Dresden Germany; c Center for Advanced Systems Understanding, CASUS Untermarkt 20 02826 Görlitz Germany; d Department of Chemistry and, ibs for Nanomedicine, Yonsei University Seodaemun-gu Seoul 120-749 Republic of Korea

## Abstract

Exotic band features, such as Dirac cones and flat bands, arise directly from the lattice symmetry of materials. The *Lieb* lattice is one of the most intriguing topologies, because it possesses both Dirac cones and flat bands which intersect at the Fermi level. However, the synthesis of *Lieb* lattice materials remains a challenging task. Here, we explore two-dimensional polymers (2DPs) derived from zinc-phthalocyanine (ZnPc) building blocks with a square lattice (*sql*) as potential electronic *Lieb* lattice materials. By systematically varying the linker length (ZnPc-*x*P), we found that some ZnPc-*x*P exhibit a characteristic *Lieb* lattice band structure. Interestingly though, *fes* bands are also observed in ZnPc-*x*P. The coexistence of *fes* and *Lieb* in *sql* 2DPs challenges the conventional perception of the structure–electronic structure relationship. In addition, we show that manipulation of the Fermi level, achieved by electron removal or atom substitution, effectively preserves the unique characteristics of *Lieb* bands. The *Lieb* Dirac bands of ZnPc-4P shows a non-zero Chern number. Our discoveries provide a fresh perspective on 2DPs and redefine the search for *Lieb* lattice materials into a well-defined chemical synthesis task.

## Abstract 2

ZnPc-*x*P polymers with square pores are Chern insulators and exhibit *Lieb* lattice band features, *i.e.*, Dirac cones intersecting a flat band at the Fermi level. Manipulating the Fermi level preserves these distinctive *Lieb* bands.

## Graphical abstract

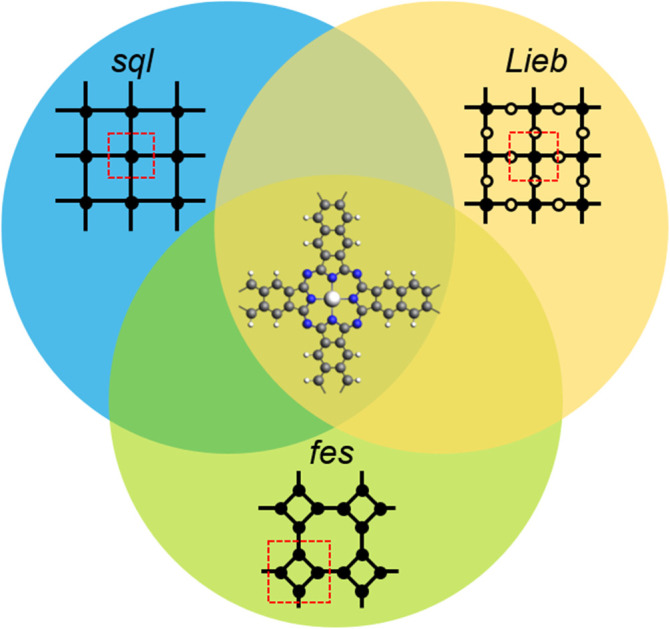

## Introduction

Exotic electronic structures, exemplified by Dirac cones and flat bands, have emerged as a focal point in contemporary research due to their unique electronic properties, including exotic charge carrier mobilities and the induction of topological effects.^1,2^ An ample example of these exotic electronic structures is the Dirac cones in a honeycomb (*hcb*) lattice, which were first predicted theoretically and only later gained importance with the discovery of graphene.^3,4^ Since then, graphene has found many applications in electronic devices, high-speed transistors, spintronics, photonics and optoelectronics.^5^ All these applications are possible thanks to the Dirac cone, characterized by crossing bands with linear dispersion intersecting at the K point of the Brillouin zone. This implies the existence of massless electrons from a non-relativistic perspective, consequently leading to exceedingly high electron mobility and topological effects.^4,6^ On the other hand, flat (dispersionless) bands are characterized by electrons with extraordinarily large effective masses and energies that are independent of the carrier momentum.^2,7,8^ Partially filled flat bands can then result in novel phases of matter, such as superconductivity, magnetism, and metal–insulator transitions.^9^

**Fig. 1:**
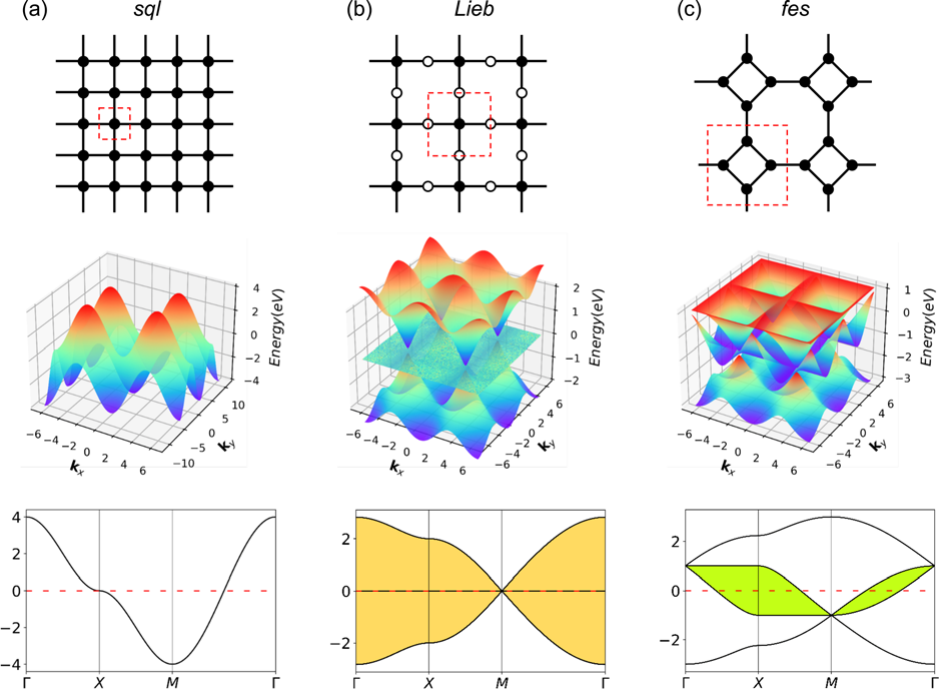
Schematic models and band structures of (a) *sql*, (b) *Lieb* and (c) *fes* lattices in the tight-biding model, considering only 1st-neighbor interactions, the next-nearest neighbor interactions are set to zero. The red dashed lines indicate the unit cells. Yellow and green areas indicate *Lieb* and *fes* bands, respectively.

The relationship between the *hcb* lattice and Dirac cones can be generalized to a statement that electronic structure features arise directly from the lattice symmetry of the materials.^10^ Notably, many of the distinctive electronic features are shared between very different lattices, with Dirac cones appearing, *e.g.*, in kagome (*kgm*), *hcb*, *fes*^11^ and *Lieb*^12^ lattices, and flat bands, *e.g.*, in *kgm* and *Lieb*.^10,13^ Among these, the *Lieb* lattice signature electronic structure is a very interesting one because it contains flat bands exactly crossing the Dirac cone (denoted as “*Lieb* bands” in the remaining paper) (Fig. 1).^7^ Another related lattice derived from square symmetry is the *fes* lattice, containing square and octahedral pores (Fig. 1c). Its characteristic band structure (denoted as *fes* bands in the remaining manuscript) has two high-symmetry crossing points (Γ and M), with one locally flat band intersecting a Dirac cone.^10,14^

*Lieb* bands may be interesting from the viewpoint of electronic topology due to the effect of spin–orbit coupling (SOC), since the Dirac bands in an ideal *Lieb* lattice (the corner and edge sites are in the same energy, dE = 0) can have a non-zero Chern number and the contact between them and the flat bands is protected by the real-space topology. When dE ≠ 0, where the corner and edge sites are in different energies, the flat band shows a non-zero Chern number, while one of the Dirac bands shows a zero Chern number with a band gap between it and the flat band being opened.^10,15,16^ Theoretical predictions using the Tight-Binding (TB) model show that *Lieb* bands require ideal lattice symmetry and strict conditions on state energies.^15,17,18^ Because of these very strict criteria, the electronic *Lieb* lattice has rarely been achieved experimentally.

So far, the *Lieb* lattice has been investigated mainly by using optical lattices,^19^ or by surface deposition of small molecules.^20^ A recently synthesized two-dimensional (2D) sp^2^ carbon-conjugated covalent organic framework (COF)^21^ was theoretically demonstrated to have *Lieb* bands.^15,18^ Soon after, the ZnPc-1P polymer, an analogue to the experimentally achievable FePc polymer,^22^ with zinc-phthalocyanine as the lattice center and benzene ring as the linker, was predicted to have *Lieb* bands, which remain topologically non-trivial after chemical substitution or physical strain engineering.^17^ However, while the ZnPc-1P band structure was identified as a *Lieb* lattice in the original paper, it much more resembles that of the *fes* lattice, which has been in depth studied in ref. 14 and 23.

Zhou *et al.* reported the intriguing finding that structural and electronic lattices can differ as is the case of hexagonal (*hxl*) sd^2^ graphene displaying a *kgm* electronic structure.^24^ While similar results have been found in other materials,^25^ commonly between *hxl* and *kgm* lattices, to date there has been no exploration of the *sql vs. Lieb*/*fes* scenario.

**Fig. 2:**
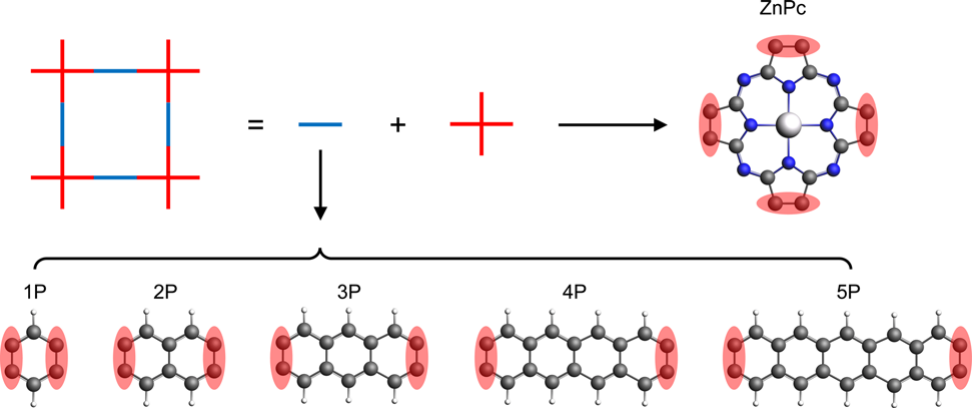
Schematic representation of the ZnPc-*x*P 2DP series structures. The red shaded area indicates the shared atoms between the center and linker molecules.

In this study, we have investigated a series of hypothetical two-dimensional polymer (2DP) structures derived from the zinc-phthalocyanine (ZnPc) 2DP as model *sql* polymers. These derivatives, denoted as ZnPc-*x*P, feature linkers with varying lengths, where *x* represents the number of aromatic rings in the linker (ranging from 1 to 5, Fig. 2). Interestingly, the band structure of the ZnPc-*x*P 2DPs, while structurally having a simple *sql* lattice, exhibits an evolution from *fes* bands to *Lieb* bands, depending on the linker chain length. In particular, the *Lieb* bands of the ZnPc-4P material are in perfect agreement with the TB model of the *Lieb* lattice, including their topological properties. We have also shown that the features of the *Lieb* bands are preserved when the Fermi level is shifted by both simple electron removal or atom substitution, thereby transforming the challenge of *Lieb* lattice search into a well-defined chemical synthesis task.

## Results

**Fig. 3:**
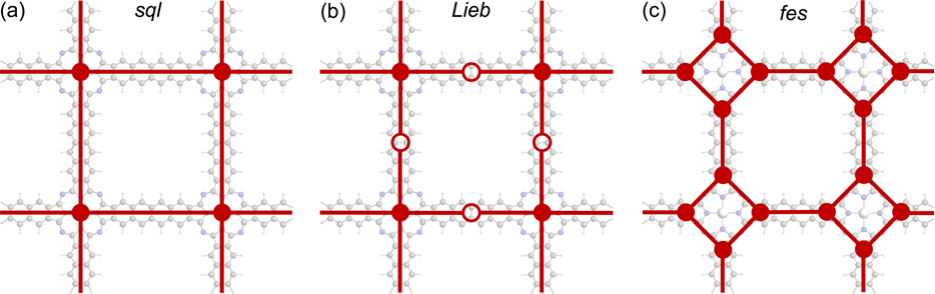
Possible topologies (a) *sql*, (b) *Lieb* and (c) *fes* lattices projectable on the ZnPc-*x*P 2DPs. ZnPc-4P is shown as an example.

The basic building units of the 2DPs of interest in this study are the ZnPc molecule and acenes (benzene, naphthalene, anthracene, tetracene, and pentacene, denoted as 1P, 2P, 3P, 4P, and 5P, respectively). These building blocks assemble into flat 2D sheets with square pores, forming ZnPc-*x*P 2DPs, where *x* = 1, 2, …, 5 represents the number of aromatic rings in the linker. The ZnPc-*x*P 2DP family, although only hypothetical structures, are mechanically stable based on their phonon dispersion (Fig. SI-1†) and represent a very interesting case study into the lattice structure and electronic properties relationship. From the structural point of view, common in experimental/synthetic materials community, they would be considered as *sql*. However, in a deeper look, *Lieb* and *fes* lattices can also be formally projected on the ZnPc-*x*P geometries (Fig. 3). This is confirmed by the reported band structure of ZnPc-1P, 2P and 3P by Pham *et al.*,^26^ and 4P, 5P by Raptakis *et al.*,^27^ which show emerging *Lieb* bands. In order to explain this interesting finding, herein we investigate the relationship between ZnPc-*x*P 2DP structures and electronic properties, particularly considering them as possible materials possessing *Lieb* bands.

**Fig. 4:**
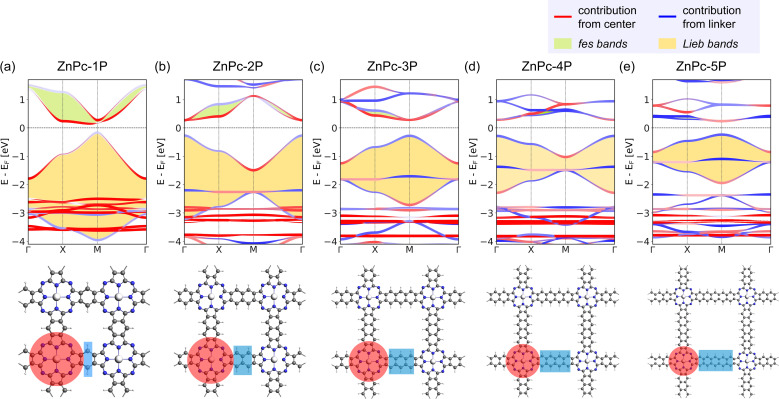
Band structure and the top view of the ZnPc-*x*P 2DPs of (a) ZnPc-1P, (b) ZnPc-2P, (c) ZnPc-3P, (d) ZnPc-4P, and (e) ZnPc-5P. The red and blue bands indicate the orbital contribution from the center/linker, respectively, which are also highlighted in the crystal structure using the same color scheme. *fes* band and *Lieb* band features are highlighted with green and yellow backgrounds in the band structures.

Indeed, the band structures of ZnPc-*x*P 2DPs shown in Fig. 4 include both *fes* band and *Lieb* band features. The *fes* bands are obviously present in the band structure of ZnPc-1P 2DP, the feature becomes weaker as the linker length increases, as indicated by the green area. The *Lieb* bands can also be observed below the Fermi level (set to 0) with a distorted flat band located at about −2.5 eV. With increasing linker length, the (almost) flat band gradually approaches the Fermi level, as indicated by the yellow area in Fig. 4. It is noteworthy that the position of the Dirac cone in ZnPc-*x*P 2DPs alternates between the Γ point for *x* = 1, 3, 5, and M point for *x* = 2, 4 (Fig. 4) while keeping all features of *Lieb* bands intact. This behavior of the 2DP electronic structures has recently been reported in the same material by Raptakis *et al.*^27^ and attributed to the parity of the symmetry. Upon further investigation, we found that the same behavior can be observed in other classes of square polymers, as shown in the model structures in Fig. SI-2.†

The contribution of the center/linker to the band structure of ZnPc-*x*P 2DPs is shown in Fig. 4 (center defined as a porphyrin molecule equivalent and linker as the connector in between). The flat band contributes from both the linker and the center, while the bands forming the Dirac cone originate from both the center and the linker. The *fes* bands in ZnPc-1P are dominated by the center with a partial contribution from the linker. The contribution varies depending on the definition of the center/linker region in the 2DP structures, as shown in Fig. SI-3,† but shows the same trend: as the linker length increases, the contribution of the linker to the Dirac cone bands gradually increases, while the *fes* lattice features become less pronounced.

**Fig. 5:**
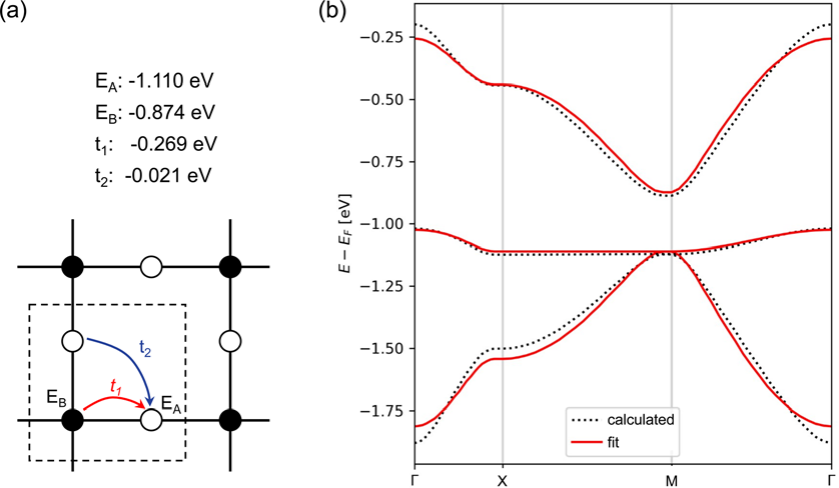
(a) *Lieb* lattice with four parameters, the center site *E*_A_, the corner site *E*_B_, the nearest neighbor hopping *t*_1_ and the next-nearest neighbor hopping *t*_2_. (b) The TB fitting of DFT calculated band structures.

To further verify that ZnPc-*x*P 2DPs can be regarded electronically as a *Lieb* lattice, we have fitted the band structure to a TB model of a *Lieb* lattice to reproduce the electronic structure of ZnPc-4P. The TB model has four parameters: on-site energies for the edge and center sites, and hopping parameters amongst the nearest neighbors and next-nearest neighbors. The parameters of the model were optimized to fit the *Lieb* bands of ZnPc-4P. Our analysis shows an excellent match between the band structures from the TB model and the bands from full ZnPc-4P (Fig. 5).

**Fig. 6:**
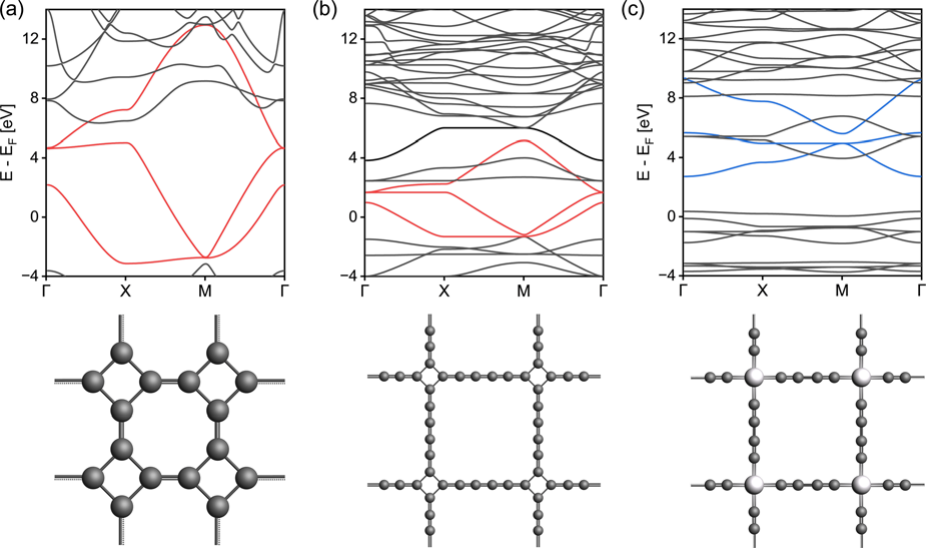
Band structures and the top view of model structures connected by (a) T-graphene, (b) T-graphtriyne, and (c) Zn-diyne. Features of *fes* and *Lieb* in the band are colored in red and blue, respectively.

In order to understand how the electronic topology originates from the atomistic structure, we have constructed three simplistic, full-atomic hypothetical carbon allotrope models featuring *fes* and *sql*/*Lieb* lattices similar to the TB lattice models. The first model (Fig. 6a), T-graphene,^28^ constitutes exactly the *fes* lattice, and produces nearly ideal *fes* bands. The electronic features of the *fes* lattice are preserved in T-graphtriyne^29^ (Fig. 6b), which incorporates an extended linker between the rhombic nodes. If the rhombic center of T-graphtriyne is substituted with a single Zn atom forming Zn-diyne (Fig. 6c), which would traditionally be considered an *sql* lattice, distorted *Lieb* bands emerge instead of the *sql* bands.^10^ These simple models confirm that the *sql* TB model is too simplistic to describe square-pore 2DP systems, since even a simple *D*_4h_ symmetry structure as in Fig. 6c contains *Lieb* bands. The reason for this is that the electronic structure is defined by the topology of the scalar field of electron density rather than by simple geometry, which means that the lattice of the material cannot be simply mapped by its atomistic structure.

We have evaluated the topological properties of the ZnPc-*x*P materials by calculating their Chern numbers, taking into account SOC. The Chern numbers of the three *Lieb* bands are (−1 1 0) from the bottom band to the top band. The fitted TB model for ZnPc-4P shows the same Chern numbers. The topological properties often depend on the strength of the SOC effect structure, which can be modulated, *e.g.*, by incorporating heavy metal atoms into the structure. We have investigated this on the fitted TB model of ZnPc-4P by scanning the effect of artificially set SOC values. Under heavy SOC effects, robust edge states can emerge in the electronic structure (Fig. SI-4 and 5†).

**Fig. 7:**
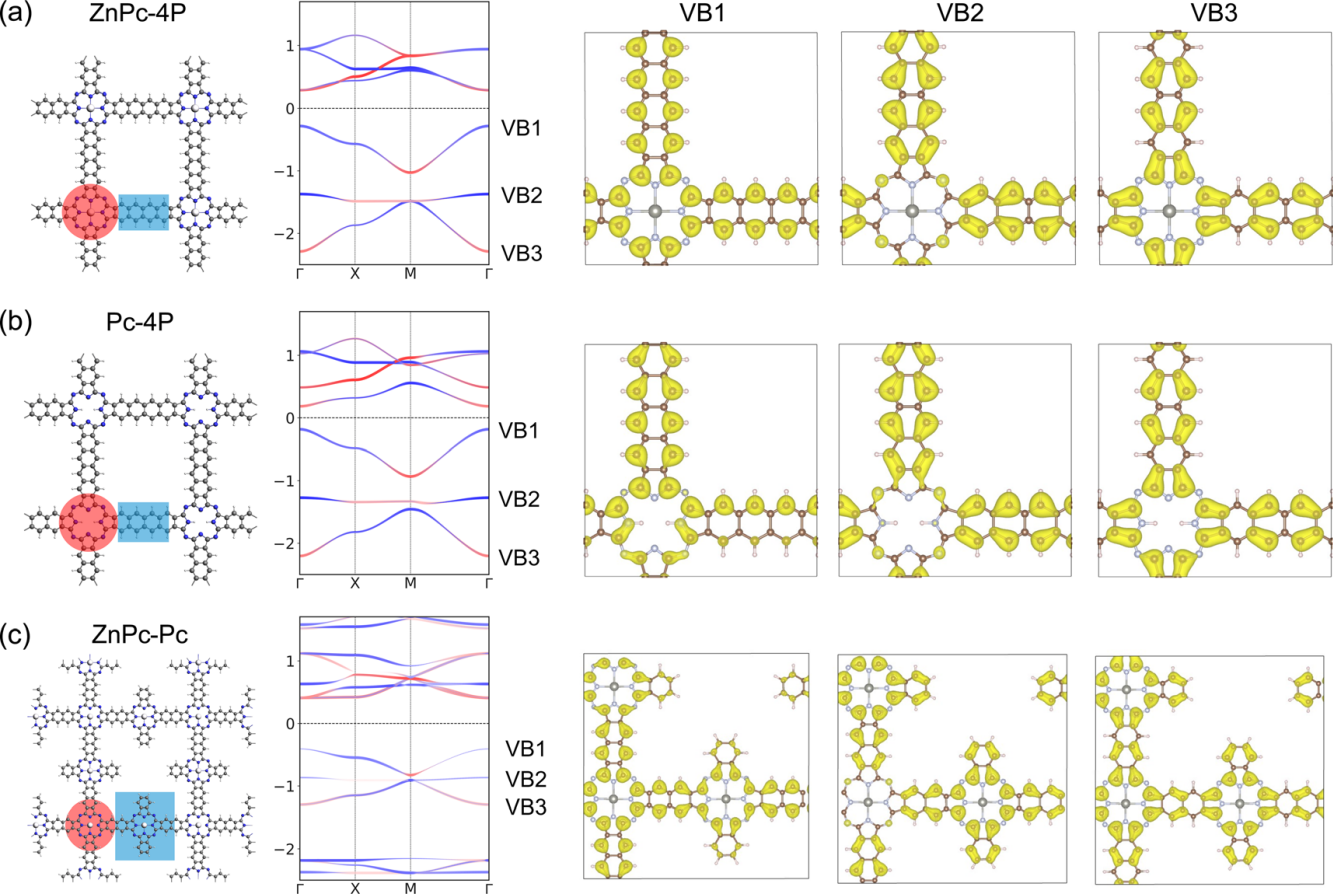
Structure, band structure, and charge density distribution of the upper three VB bands of (a) ZnPc-4P with Zn atom, (b) Pc-4P without Zn atom, (c) ZnPc–ZnPc 2DP. The red and blue bands indicate the orbital contribution from the center/linker, with the center–linker partitioning highlighted in the crystal structure using the same color scheme.

The primary limitation of the studied 2DPs lies in the positioning of the *Lieb* bands below the Fermi level. Achieving non-trivial structural properties in these bands thus requires shifting the Fermi level while preserving the *Lieb* bands. To address this, we first conducted an analysis of the atom contributions to the charge density within the *Lieb* bands (VB1, VB2, and VB3) using ZnPc-4P 2DP as a model system (Fig. 7a and SI-6†). The primary contribution to the *Lieb* bands comes from the linker (tetracene) π-electrons, which contribute to all VB1-3, although the carbons in the phthalocyanine center also partially contribute to the Dirac bands VB1 and VB3 (Fig. 7).

The contribution of the metal atom to the *Lieb* bands is only minimal. This is also confirmed using a structure without a metal atom in the phthalocyanine center (Pc-4P), which gives almost identical *Lieb* bands to ZnPc-4P (Fig. 7). However, the *fes* features in conduction bands collapse after the removal of the metal atom.

Additionally, we have designed a hypothetical reference porphyrin polymer characterized by an “ideal atomic *Lieb* lattice” structure ZnPc–ZnPc 2DP (Fig. 7c and SI-7†). Both *fes* features in conduction bands and *Lieb* bands in valence bands are present with only minor changes. *Lieb* bands again contain the full π-system of the linker, despite its bigger size. This suggests that the well-ordered conjugated π-system is important for achieving high quality *Lieb* electronic materials.

**Fig. 8:**
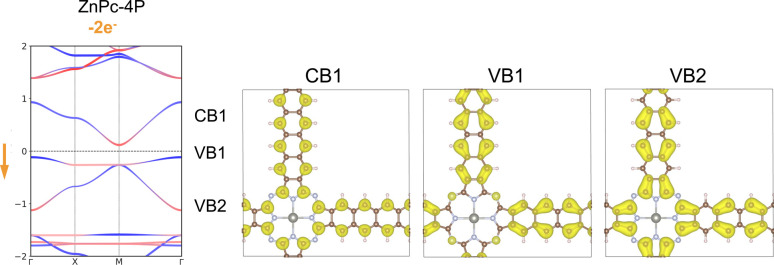
Band structure and charge density distribution of the three *Lieb* bands of ZnPc-4P with two electrons removed (two holes introduced) per unit-cell. The red and blue bands indicate the orbital contribution from the center/linker.

We have investigated the modulation of the Fermi level position by two different methods: the direct removal of electrons from the system and atomic substitution.^30^ The removal of two electrons per unit cell effectively shifts the Fermi level toward the flat band. This adjustment results in a slight increase in the dispersion of the flat band, but leaves the fundamental *Lieb* bands intact (Fig. 8). The charge density contributions from the *Lieb* bands show identical features to those observed in the pristine structure. The Chern number of flat band VB1 is −1, while it is 0 for Dirac bands.

**Fig. 9:**
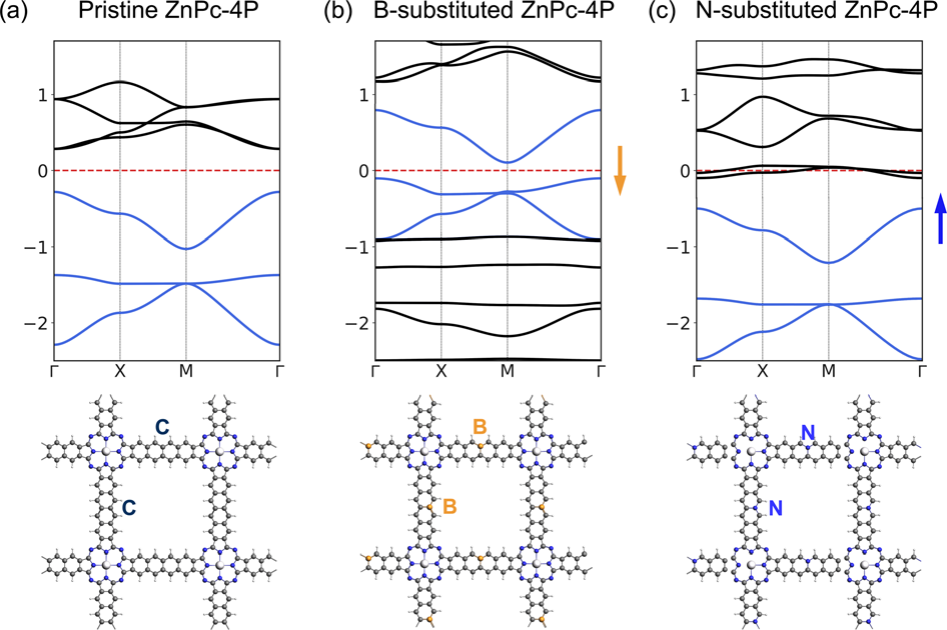
Band structures, and top view of the structures of (a) pristine (b) *B*-substituted, (c) *N*-substituted ZnPc-4P. *Lieb* bands are colored in blue. The arrow indicates the Fermi level shift direction.

The charge density analysis of VB1-3 (Fig. 7) shows that in order to access the flat band, electrons should be removed from the aromatic system of the linkers, especially in the edge atoms of the linker. We have tested replacing individual carbons (2 atoms per UC, one per linker) in the linker with boron atoms, shifting the Fermi level to the flat band; and with nitrogen, shifting the Fermi level to the *fes* bands (Fig. 9). In the B-substituted structure, the Fermi level shifts to the *Lieb* bands, and most of the band structure features are preserved, although the dispersion of the “flat band” is stronger. The two Dirac bands of the *Lieb* bands show a non-zero Chern number. In the *N*-substituted structure, the *Lieb* bands are also preserved. These substituted model structures, although not easily achieved chemically, could provide a general guide how *Lieb* bands could be accessed in other square pore 2DPs with more suitable structures for substitution.

## Conclusions

We have investigated the ZnPc-*x*P 2DPs as a model system for electronic structure topology of square pore 2DPs. While these materials are traditionally considered to have an *sql* topology well known from TB models, our results challenge this oversimplified view. We found that ZnPc-*x*P 2DPs exhibit both *fes* bands and *Lieb* bands while completely lacking the expected *sql* features. This is because the electronic structure of these materials is governed by the topology of the electron density, rather than by simple atomic geometry. Furthermore, the flat band and bottom Dirac band of the *Lieb* bands possess a non-zero Chern number. Unfortunately, the *Lieb* bands in the ZnPc-*x*P 2DPs are located below the Fermi level, as well as in most other model materials studied here. However, we have shown that shifting the Fermi level by controlling the number of electrons in the system *via* gating or substitution preserves the *Lieb* bands including their topological character. The challenge of finding *Lieb* lattice structures thus turns into a well-defined chemical synthesis task. This opens up the possibility of designing materials with unique properties, such as Chen semiconductors. We hope that our work will stimulate further experimental exploration of *Lieb*-lattice-based topological materials.

## Methods

The geometries were optimized using the self-consistent-charge density functional based tight binding (SCC-DFTB)^31^ method as implemented in the Amsterdam Modelling Suite (AMS) ADF 2019.^32^ The 3ob-3-1 parameter set^33^ was used for systems with X–Y element pair interaction (X, Y = C, H, Zn), and the matsci-0-3 parameter set was applied for systems including boron.^34^ Band structure calculations were performed employing FIH-aims with a TIER1 basis set with a 4 × 4 × 1 k-mesh grid,^35^ using DFT with the Heyd–Scuseria–Ernzerhof 2006 functional.^36^. The key parameter “tight” regarding computational accuracy was used to control all integration grids, and the accuracy of the Hartree potential. The scalar relativistic effects were involved by using the atomic zeroth order regular approximation (ZORA) level of theory. Tight-binding models were built using PythTB,^37^ under consideration of both nearest-neighbor and next-nearest neighbor interactions, as well as SOC. The topological properties were simulated based on the Haldane model. The Berry curvature and the intrinsic anomalous Hall conductivity were performed using the WANNIER90 package.^38^ Calculations of edge states and the Chern number were carried out using the WannierTools package.^39^ SOC and spin polarization were taken into account in the topological calculations. Phonon dispersions were calculated using Phonopy & DFTB+ with a 4 × 4 × 1 supercell.^40^

## Data availability

The geometries and corresponding band structures for all systems investigated in this work are available at the NOMAD repository as a dataset under DOI: https://doi.org/10.17172/NOMAD/2023.11.26-1.

## Author contributions

Y. Z. and S. Z. performed calculations and theoretical analysis. M. P. and T. H. supervised the study. Y. Z. wrote the manuscript with contributions of all authors, and all authors have given approval for the final version of the manuscript.

## Conflicts of interest

There are no conflicts to declare.

## Supplementary Material

SC-015-D3SC06367D-s001
