# Supplementary material for: Electronic Lieb lattice signatures embedded in two-dimensional polymers with a square lattice
Source: Chem Sci. 2024 Mar 12;15(15):5757–63. doi: 10.1039/d3sc06367d (PMC11023029; doi:10.1039/d3sc06367d)
Supplement: SC-015-D3SC06367D-s001 [file SC-015-D3SC06367D-s001.pdf]

Electronic Supplementary Material (ESI) for Chemical Science.
This journal is © The Royal Society of Chemistry 2024

Electronic Lieb lattice signatures embedded in two-dimensional polymers with square lattice

Yingying Zhang,[abc] Shuangjie Zhao,[a] Miroslav Položij,[abc] Thomas Heine*[abcd]

[a.] Chair of Theoretical Chemistry, Technische Universität Dresden, Bergstrasse 66, 01069 Dresden, Germany

[b.] Helmholtz-Zentrum Dresden-Rossendorf, Bautzner Landstr. 400, 01328 Dresden, Germany

[c.] Center for Advanced Systems Understanding, CASUS, Untermarkt 20, 02826 Görlitz, Germany

[d.] Department of Chemistry and ibs for nanomedicine, Yonsei University, Seodaemun-gu, Seoul 120-749, Republic of Korean.

E-mail: thomas.heine@tu-dresden.de

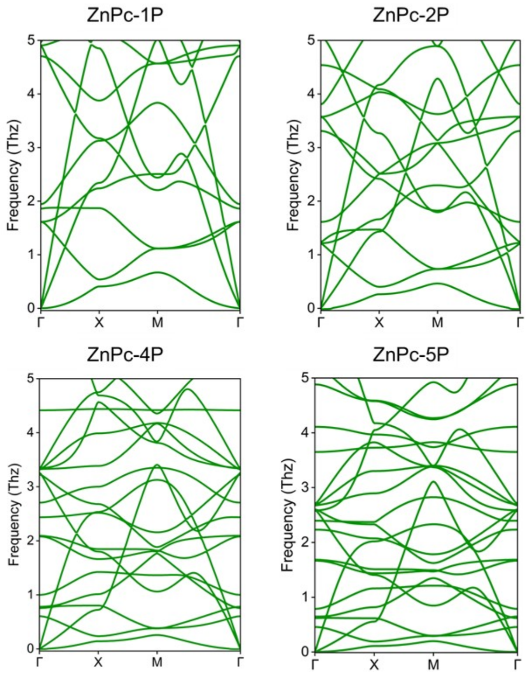

Figure SI-1. Phonon dispersions of ZnPc-*x*P 2DPs.

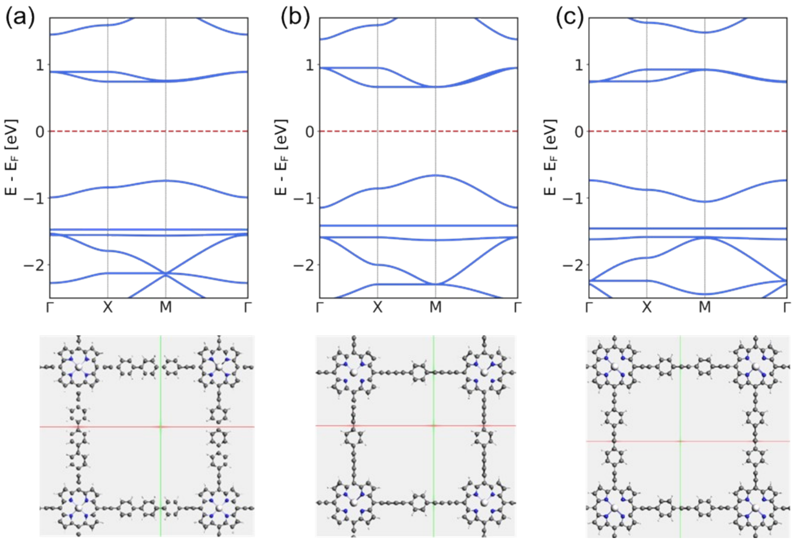

Figure SI-2. Band structure and the top view of the ZnPc-*x*P COF, connected with porphyrin and phenyl group. (a) ZnPP-3P COF, (b) ZnPP-1P COF, and (c) ZnPP-2P COF, showing the parity of the Dirac cone position, altered between M and Γ.

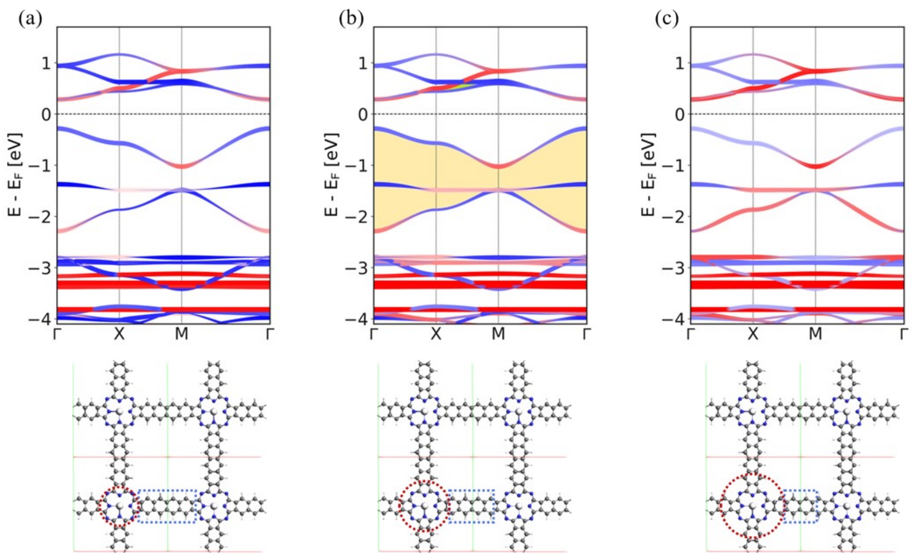

Figure SI-3. Band structure and the top view of the ZnPc-4P COF in different boarder position between center/linker. The red and blue bands indicate the orbital contribution from center and linker, which are highlighted with the same color in the crystal structure.

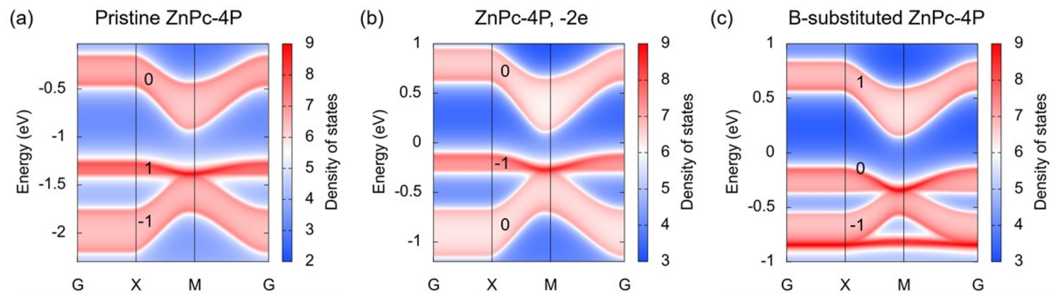

Figure SI-4. Edge states and Chern number of (a) pristine ZnPc-4P, (b) ZnPc-4P with two electrons removed (two holes introduced) per unit-cell, (c) B-substituted ZnPc-4P.

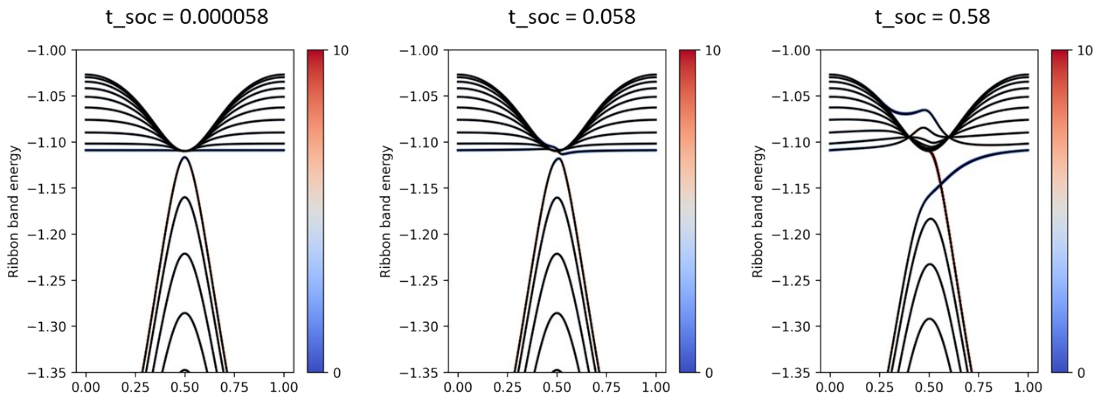

Figure SI-5. Edge states of the fitted TB model of ZnPc-4P at different degrees of spin-orbit coupling (SOC).

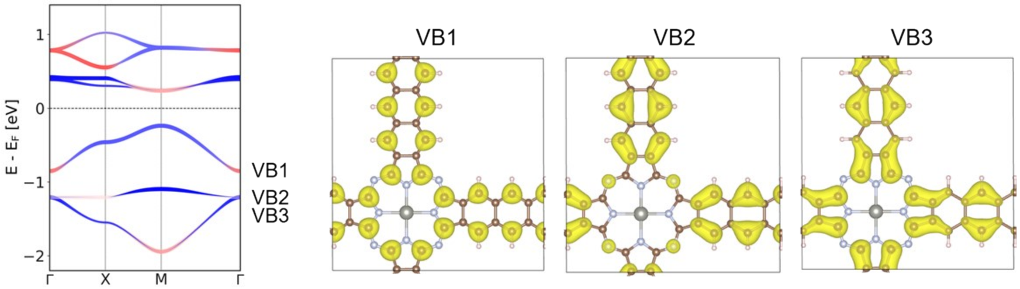

Figure SI-6. Band structure, and charge density distribution of the top three VB bands of ZnPc-5Bz.

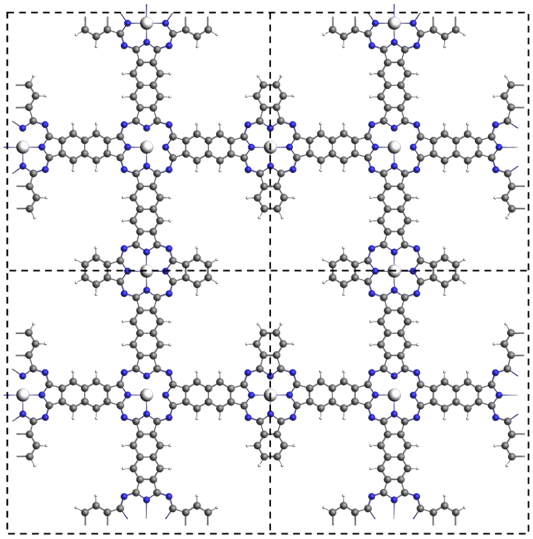

Figure SI-7. Top view of ZnPc-ZnPc COF.
